# Retraction Note: csi-miR-96-5p delivered by *Clonorchis sinensis* extracellular vesicles promotes intrahepatic cholangiocarcinoma proliferation and migration via the ferroptosis-related PTEN/SLC7A11/GPX4 axis

**DOI:** 10.1186/s13071-024-06408-0

**Published:** 2024-07-18

**Authors:** Li-Jia Wen, Ji-Gang Yin, Yong-Xin Wang, Kai Liu, Ji-Xue Zhao

**Affiliations:** 1 Department of Pediatric Surgery, The First Hospital of Jilin University, Changchun, 130021 Jilin China; 2 State Key Laboratory for Diagnosis and Treatment of Severe Zoonotic Infectious Diseases, Key Laboratory for Zoonosis Research of the Ministry of Education, Institute of Zoonosis, College of Veterinary Medicine, Jilin University, Changchun, 130062 Jilin China; 3 Department of Hepatobiliary and Pancreatic Surgery, General Surgery Center, The First Hospital of Jilin University, Changchun, 130021 Jilin China

**Retraction: Parasites & Vectors (2023) 16:465** 10.1186/s13071-023-06075-7

The Editors-in-Chief have retracted this article because concerns were raised over the presentation of the figures; this includes undeclared splicing of western blots in Figs. 2g and 5g, 5h, 6h, 6i and the alteration of contrast and resizing of these images. Western blot images in Figs. 2, 5 and 6, as well as cell transfection images were replaced between acceptance and publication, and were therefore not subjected to peer review. The authors have stated they will perform repeat experiments and a revised manuscript may be submitted for reconsideration.

All authors agree to this retraction.

